# Clinical predictors and RT-PCR profile of prolonged viral shedding in patients with SARS-CoV-2 Omicron variant in Shanghai: A retrospective observational study

**DOI:** 10.3389/fpubh.2022.1015811

**Published:** 2022-10-24

**Authors:** Wen Zhang, Shuang Zhou, Gang Wang, Min Cao, Ding Sun, Wei Lu, Li Shi, Yong Guo, Xiangru Xu, Yuting Pu, Caiyu Chen, Hongqiang Yang, Yuting Sun, Hongyi Hu, Bangjiang Fang

**Affiliations:** ^1^Department of Emergency, Longhua Hospital, Shanghai University of Traditional Chinese Medicine, Shanghai, China; ^2^School of Acupuncture-Moxibustion and Tuina, Shanghai University of Traditional Chinese Medicine, Shanghai, China; ^3^Department of Critical Care Medicine, The Second Affiliated Hospital of Xi'an Jiaotong University, Xi'an, China; ^4^Department of Rheumatology, Longhua Hospital, Shanghai University of Traditional Chinese Medicine, Shanghai, China; ^5^Department of Nursing, Longhua Hospital, Shanghai University of Traditional Chinese Medicine, Shanghai, China; ^6^Department of Pediatrics, Longhua Hospital, Shanghai University of Traditional Chinese Medicine, Shanghai, China; ^7^Shanghai Jiao Tong University Affiliated Sixth People's Hospital, Shanghai, China; ^8^Longhua Hospital, Shanghai University of Traditional Chinese Medicine, Shanghai, China; ^9^Institute of Emergency and Critical Care Medicine, Shanghai University of Traditional Chinese Medicine, Shanghai, China

**Keywords:** prolonged SARS-CoV-2 RNA shedding, SARS-CoV-2 Omicron variant, predictors, CT values, ORF 1ab gene

## Abstract

**Objective:**

To evaluate determinants of prolonged viral RNA shedding in hospitalized patients with severe acute respiratory syndrome coronavirus 2 (SARS-CoV-2) Omicron variant infection.

**Materials and methods:**

Hospitalized patients tested SARS-CoV-2 positive by nasopharyngeal real-time reverse transcriptase-polymerase chain reaction (RT-PCR) were included in the single-center, retrospective study. Patients were divided into 2 groups according to the timing of viral clearance (≤ 8 days, “early clearance” and ≥15 days, “late clearance”).

**Results:**

4,084 patients were included in the study (1,023 late clearance, 3,061 early clearance), with median age of 50 years and a higher proportion (61.4%) of male. Univariate analyses showed that comorbidities (including hypertension, diabetes, and coronary heart disease), receiving vaccine, the number of vaccinations, cycle threshold (Ct) open reading frame 1ab (ORF 1ab), and nucleocapsid protein (N) gene values on admission were associated with late viral clearance. In the multivariable analysis, the number of vaccinations (*P* = 0.010) and Ct ORF 1ab gene (*P* < 0.001) values on admission were significantly associated with late viral clearance. Generalized Estimating Equations (GEE) analysis showed that the Ct value of ORF 1ab gene and N gene remained unchanged within 3 days, and showed progressively higher values with increasing days during late viral RNA clearance.

**Conclusion:**

The number of vaccinations and Ct values of ORF 1ab gene were independently associated with a prolonged SARS-CoV-2 RNA shedding.

## Introduction

The severe acute respiratory syndrome coronavirus 2 (SARS-CoV-2) Omicron variant has been continuing to spread throughout the globe since it was first confirmed on November 9, 2021, reported to WHO from South Africa on November 24, 2021. As of May 9, 2022, the number of confirmed COVID-19 cases are over 526 million globally, including more than six million deaths (1). Since late February 2022, SARS-CoV-2 Omicron variant had appeared rapidly in Shanghai, China, and subsequently became the catalyst for COVID-19 outbreaks. According to reports by Shanghai Municipal Health Commission, as of May 4, 2022, 59,3336 cases have been identified, including 53,8450 cases of asymptomatic infection (2, 3). The viral genomes in Shanghai were clustered into the SARS-CoV-2 BA.2.2 sub-lineage. More than thirty mutations found in the spike proteins of Omicron variant with enhanced transmissibility and immune escape, which is correlated with the recent exponential growth in case counts (4, 5). According to the newest National Health Commission of China report, the Omicron variant was first identified in China on December 9, 2021, and subsequently propels the pandemic in Shanghai (6). Since March 2022, Shanghai have adopted mobile cabin hospitals and appointed hospitals to isolate and treat COVID-19 patients.

Since then, more than 50,000 confirmed cases have recovered and been discharged from hospital. However, some patients with prolonged viral shedding were reported SARS-CoV-2 positive by real-time reverse transcriptase-polymerase chain reaction (RT-PCR) again after discontinuation of quarantine, which increased the complexity of disease control and has attracted widespread concerns (7). To date, several studies, mainly during the beginning of the epidemic, have been performed to investigate the clinical characteristics and virologic course of persistently positive patients. Xu et al. evaluate 113 patients who were admitted between January 13 and February 19, 2020 (8). They found that delayed admission to hospital, male, and invasive mechanical ventilation were independent factors associated with the duration of SARS-CoV-2 RNA shedding. Zhao et al. (9) retrospectively analyzed 104 patients with COVID-19 admitted to the isolation wards of our hospital from January 19 to March 18, 2020. The result showed that the clearance of viral RNA in sputum was delayed in severe COVID-19 patients, especially in those with a lower virus cycle threshold (Ct) value. Lee et al. (10) studied 1,186 asymptomatic and mildly symptomatic COVID-19 patients in South Korea. In their study, respiratory symptoms are the strongest independent predictive indicator, while age and underlying conditions were not significantly related. Hu et al. (11) supported that older age and chest tightness were independently associated with delayed clearance of SARS-CoV-2 RNA in hospitalized patient. Zamacona and his group (12) found that chronic rhinosinusitis and atopy might be associated with increased risk of prolonged viral shedding. Clinical characteristics of Omicron variant are a bit different to other variants. Takahashi et al. (13) studied virus shedding duration by analyzing clinical samples from the upper respiratory tracts of persons infected with SARS-CoV-2 Omicron variant in Japan during November 29 to December 18, 2021. The variant can be detected 10 days after diagnosis and achieved the highest proportion of virus isolates (41.7%) in 2–5 days after diagnosis. Another study analyzed the characteristics of the global perspective of Omicron, including transmission dynamic, effect on testing, and immunity (14). However, the characterization of prolonged positive cases with Omicron variant, the potential risk factors associated with persistently positive test results are still ambiguous.

According to the latest research, Omicron variant cases a mean time to nucleic acid conversion of 6.7 days (15). Our previous study with 4,264 Omicron variant patients observed detectable viral RNA in sputum or throat swabs in 8 (5–9) days after admission (16). We defined prolonged RT-PCR positivity as virus clearance time ≥15 days. Here, we conducted a retrospective study to predict risk factors influencing the persistence of SARS-CoV-2 RNA shedding, and described the clinical characteristics of prolonged positive patients and the dynamic changes of Ct PCR values with Omicron viral RNA shedding. These results may well contribute to the resolution of the current pandemic.

## Methods

### Ethical approval

The study received ethical approval from the Longhua Hospital, Shanghai University of Traditional Chinese Medicine Ethical Review Authority (DNR 2020-02150) and registered in the Chinese Clinical Trial Registry (ChiCTR2200060472).

### Study design and participants

The retrospective and matched cohort study was conducted at the makeshift hospital set up in the Shanghai New International Expo Center between April 1 and June 1, 2022. The medical institution, which was the biggest quarantine venue in Shanghai yet, had up to 15,000 beds and hospitalizing more than 40,000 patients with COVID-19 from April 1 to June 15, 2022.

All inpatients were reported to Electronic Medical Records as being diagnosed with COVID-19 confirmed by RT-PCR analysis for SARS-CoV-2 in nasopharyngeal swabs or throat swab specimens. RT-PCR has been the gold standard for COVID-19 diagnostic testing and screening. Patients were considered to be in virologic remission based on consecutive negative RT-PCR tests with an interval of at least ≥ 24 h. RT-PCR was performed using the Shanghai BioGerm Medical Technology Co., Ltd. open reading frame 1ab (ORF 1ab) and nucleocapsid protein (N) gene kit as the sole assay, following the manufacturer's instructions.

For each subject, baseline demographic, daily updates health information on symptoms experienced, RT-PCR test results, vaccines, the level of intervention, and related outcomes were collected and recorded in Electronic Medical Records.

### Procedures

We collected the data from all inpatients who were admitted as asymptomatic and symptomatic COVID-19. The diagnosis of illness at admission were assessed based on the diagnostic criteria of the ninth edition of the COVID-19 diagnosis (17). Asymptomatic carries were defined as SARS-CoV-2 positive patients without self-perceived or clinically recognizable symptoms (18). Symptomatic cases have at least one symptom, such as fever, sore throat, dry cough, malaise, and body aches or nausea, vomiting, abdominal pain, and loose stools. Prolonged viral shedding (late clearance group) was defined as a positive RT-PCR result for ≥ 15 days, as late clearance group. We selected a control population consisting in sex, ages and wards-matched COVID-19 patients (early clearance group) who turned RT-PCR negative in ≤ 8 days after admission. Exclusion criteria included (1) viral RNA clearance occurred within 8–15 days, (2) no positive swab during their hospital stay, (3) be transferred to other hospitals, and (4) be admitted as severe/critical COVID-19.

**Figure 1:**
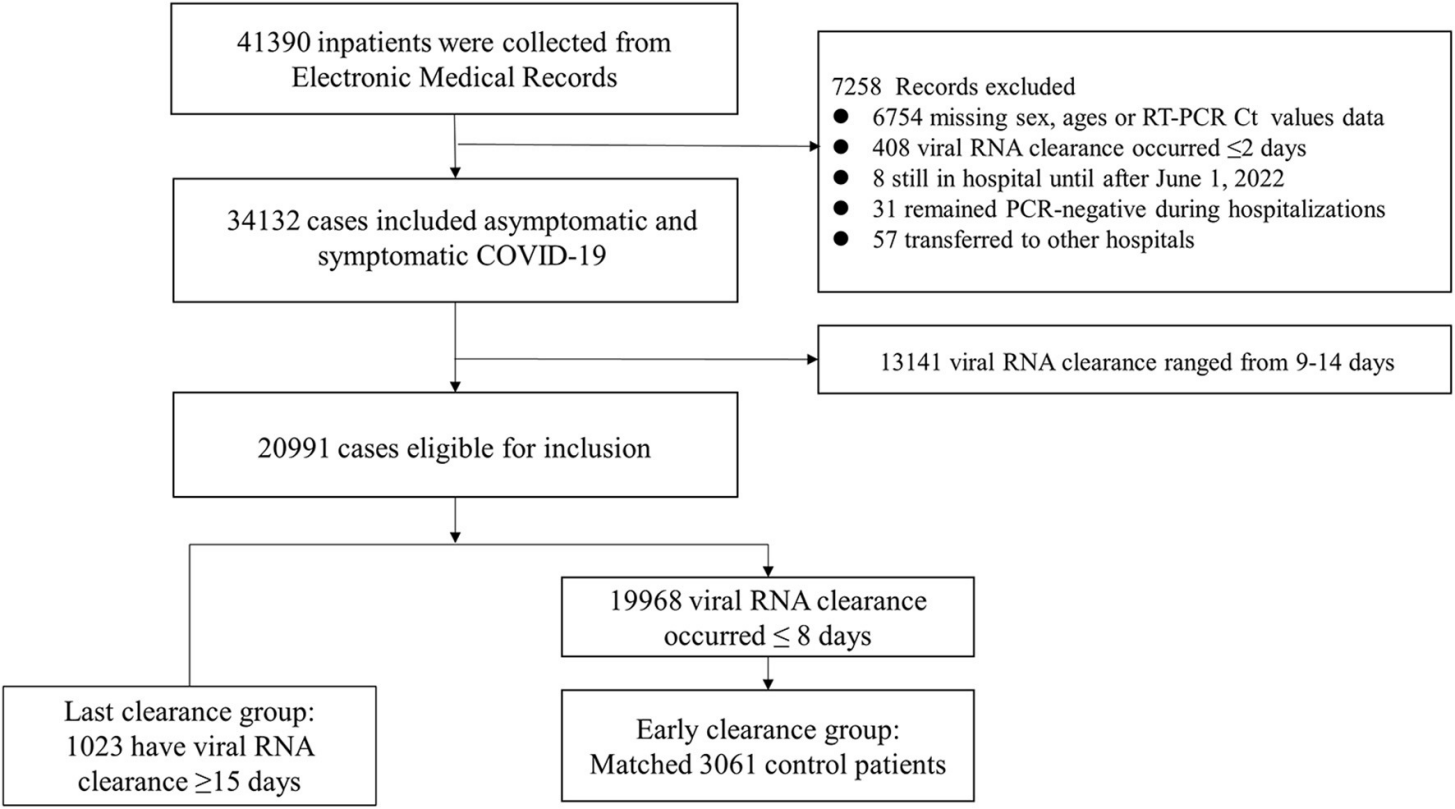
Flow chart of study.

A total of 41,390 patients' data were obtained to explore the demographic characteristics, clinical features, and risk factors for prolonged viral shedding. Of those, 6,754 cases with missing data on sex, ages, or RT-PCR cycle Ct values, 408 patients with viral RNA clearance occurred less than or equal to 2 days, eight patients who may not be discharged on June 1, 2022, 31 patients remained PCR-negative during hospitalizations and 57 patients transferred to other hospitals were excluded from this analysis. Ultimately, 13,141 patients with viral RNA clearance occurred range from 9 to 14 days were excluded. Among the remaining 20,991 cases, 1,023 have viral RNA clearance occurred ≥15 days, with 19,968 patients' viral RNA clearance occurred ≤ 8 days. Three control individuals were randomly matched by sex, ages and wards by Stata MP 17 for every patient with viral RNA clearance occurred ≤ 8 days. Finally, a total 4,084 hospitalized COVID-19 patients confirmed by RT-PCR nasopharyngeal swab samples testing were analyzed. The flow chart of study was showed in Figure 1.

### SARS-CoV-2 RNA detection using real-time RT-PCR

Novel Coronavirus (2019-nCoV) Nucleic Acid Detection Kit (PCR-Fluorescence Probing) (BioGerm, China), which targets amplified ORF1ab and N genes of SARS-CoV-2, was used for SARS-CoV-2 RNA detection according to the manufacturer's instructions. In brief, reaction mixtures were prepared with nucleic acid amplification reaction solution, enzyme mixture and ORF1ab/N reaction solution in 20 μl reaction tube. SARS-CoV-2 RNAs were extracted. RNA template and control solutions were separately added into reaction tube in 25 μl per reaction. The reaction conditions were as follows: reverse transcription at 50°C for 10 min; pre-denaturation at 95°C for 5 min; denaturation at 95°C for 10 s and then annealing and elongation at 55°C for 40s (40 cycles). Fluorescence monitoring were conducted at 55°C. The CT value of each target gene can be reported. If the CT value was <35, then the patient defined as a positive result.

### Data collection

We retrospectively collected the data from patients' electronic medical records, including ages, sex, basic disease, vaccine, race, symptoms, duration of viral RNA shedding, and Ct values of ORF 1ab and N genes. The date of diagnosis onset was also recorded. The first RT-PCR test took place at admission. At least three samples were obtained from each person until two consecutive negative PCR test results were obtained. The same type of sample was obtained in subsequent samples for follow-up. All patients were tested more than two consecutive negatives, and the first viral test date was used to define the duration of shedding.

### Statistical analysis

Continuous variables were expressed as medians with interquartile ranges (IQRs) and were compared by Kruskal-Wallis test. Categorical variables were expressed as number (%) and compared by chi-square (χ^2^) test or Fisher's exact test. Significant risk factors identified in univariate analyses were further analyzed by multivariate logistic regressions to identify independent risk factors associated with the prolonged duration of SARS-CoV-2 shedding. We used the stratified Generalized Estimating Equations (GEE) to compare viral clearance at different times. The dependent variable were the Ct values of ORF1ab and N gene. Linear GEE models were used for continuous outcome variables. The model allowed for the change with time to vary based on groups. We adopted the autoregressive (AR-1) covariance as the working covariance structures in the GEE estimation. All statistical analyses were performed using SPSS (version 25.0) and Stata MP (version 17) software. The significance level of the hypothesis tests was set at 0.05 (2-sided).

## Results

### Clinical characteristics of patients in this study

A total of 4,084 patients were included in the study for final analysis, and 1,023 of those had viral RNA shedding more than or equal to 15 days, and 3,061 control patients had persistent negative viral detection results less than or equal to 8 days. The median time from illness onset to hospital admission for all enrolled patients was 2 days (IQR, 1–3 days). The baseline characteristics were as summarized in the Table 1.

**Table 1:** Characteristics of included cases and controls tested for SARS-CoV-2 in Shanghai access to testing, China, April 1–June 1, 2022.

**Characteristic**	**All patients**	**Late clearance**	**Early clearance**	***P*-value**
Overall	4,084	1,023 (25.05%)	3,061 (74.95%)	
Sex				1.00
Male, *n* (%)	2,506 (61.4%)	628 (61.4%)	1,878 (61.4%)	
Female, *n* (%)	1,578 (38.6%)	395 (38.6%)	1,183 (38.6%)	
Ages (IQR)	50 (36–59)	51 (36–60)	50 (36–59)	0.341
Comes back from abroad	20 (0.5%)	6 (0.6%)	14 (0.5%)	0.608
Comorbidity, *n* (%)	834 (20.4%)	247 (24.1%)	587 (19.2%)	0.001
Hypertension	592 (14.5%)	168 (16.4%)	424 (13.9%)	0.043
Diabetes	214 (5.2%)	73 (7.1%)	141 (4.6%)	0.002
Coronary heart disease	84 (2.1%)	29 (2.8%)	55 (1.8%)	0.043
Congestive heart failure	14 (0.3%)	7 (0.7%)	7 (0.2%)	0.056
Stroke	27 (0.7%)	9 (0.9%)	18 (0.6%)	0.371
Thrombotic diseases	32 (0.8%)	11 (1.1%)	21 (0.7%)	0.305
Allergy	127 (3.1%)	28 (2.7%)	99 (3.2%)	0.468
Initial symptoms				0.836
Asymptomatic	3,277 (80.2%)	842 (82.3%)	2,435 (79.5%)	
Symptomatic	807 (19.8%)	181 (17.7%)	626 (20.5%)	
Vaccine	3,108 (76.1%)	712 (69.6%)	2,396 (78.3%)	<0.001
NO. vaccine				<0.001
Not vaccinated	976 (23.9%)	311 (30.4%)	665 (21.7%)	<0.05
Received 1 dose of vaccine	131 (3.2%)	39 (3.8%)	92 (3.0%)	>0.05
Completed 2 doses of vaccine	1,200 (29.4%)	275 (26.9%)	925 (30.2%)	<0.05
Completed 3 doses of vaccine	1,729 (42.3%)	392 (38.3%)	1,337 (43.7%)	>0.05
Completed ≥4 doses of vaccine	48 (1.2%)	6 (0.6%)	48 (1.2%)	<0.05
Vaccine manufacturer				<0.05
Sinovac Biotech Co., Ltd.	2008 (49.2%)	456 (44.6%)	1552 (77.3%)	<0.05
CanSino Biologics Inc	62 (1.5%)	19 (1.9%)	43 (1.4%)	>0.05
Anhui Zhifei Longcom Biopharmaceutical	22 (0.5%)	7 (0.7%)	15 (0.5%)	>0.05
China National Pharmaceutical Group Co., Ltd.	1016 (24.9%)	230 (22.6%)	786 (25.7%)	<0.05
Time from diagnosis to nucleic acid testing negative first (IQR)	3 (3–5)	8 (5–11)	3 (3–5)	<0.01
Time from diagnosis to two successive nucleic acid testing negative (IQR)	8 (6–15)	16 (15–18)	7 (6–8)	<0.01
Ct value for ORF 1ab gene at diagnosis	31.45 (25.62–36.36)	24.59 (21.03–30.00)	34.24 (29.92–37.59)	<0.001
Ct value <25	570 (14.0%)	442 (43.2%)	128 (4.2%)	<0.05
25≥ Ct value <35	1,109 (27.2%)	327 (31.9%)	783 (25.6%)	<0.05
Ct value ≥35	2,405 (58.9%)	255 (24.9%)	2,150 (70.2%)	<0.05
Ct value for N gene at diagnosis	31.04 (25.11–35.84)	24.02 (20.31–29.08)	33.96 (29.27–36.82)	<0.001
Ct value <25	588 (14.4%)	447 (43.7%)	141 (4.6%)	<0.05
25≥ Ct value <35	1,058 (25.9%)	297 (29.0%)	761 (24.9%)	<0.05
Ct value ≥35	2,438 (59.7%)	279 (27.3%)	2,159 (70.5%)	<0.05

Data are presented as No. (%) or Median (IQR). IQR, interquartile range; Ct, cycle threshold; ORF 1ab gene, open reading frame 1ab; N gene, nucleocapsid protein (N) gene.

Of 4,084 cases, the median age was 50 (IQR, 36 to 59; range, 2 to 87), 38.6% (1578/4084) were female, and 61.4% (2506/4084) were male. The most common comorbid conditions (20.4%) were hypertension (14.5%), followed by diabetes (5.2%), coronary heart disease (2.1%), congestive heart failure (0.3%), stroke (0.7%), and thrombotic diseases (0.8%). Among the patients, most of those had no symptoms (80.2%), while 19.8% cases had symptoms. A total of 3,108 (76.1%) cases received vaccine with 1 dose (3.2%), 2 dose (29.4%), 3 dose (42.3%), or more than 3 dose (1.2%). These vaccine manufacturers included Sinovac Biotech Co., Ltd. (49.2%), CanSino Biologics Inc (1.5%), Anhui Zhifei Longcom Biopharmaceutical (0.5%), China National Pharmaceutical Group Co., Ltd (24.9%). The median Ct values are 31.45 (25.62–36.36) for ORF 1ab gene, and 31.04 (25.11–35.84) for N gene (Table 1).

We grouped patients according to Ct values as low (<25), high (25≥, <35), and the negative (≥35). For ORF 1ab gene, 570 (14.0%) patients had Ct values <25, 1,109 (27.2%) patients had Ct values between 25 and 35, and 2,405 (58.9%) patients had Ct values ≥35. For N gene, 588 (14.4%) patients had Ct values <25, 1,058 (25.9%) patients had Ct values between 25 and 35, and 2,438 (59.7%) patients had Ct values ≥35 (Table 1).

No significant differences between the groups were observed for sex, ages, coming back from abroad, congestive heart failure, stroke, thrombotic diseases, allergy, the presence of symptoms. The median duration of viral shedding of these prolong RNA positive patients was 16 days (IQR, 15–18 days), while seven (6–8) in control group. The median duration of these prolonged RNA positive patients testing negative first was 3 days (IQR, 3–5 days), while eight (5–11) in control group (Table 1).

ORF 1ab gene Ct values were significantly higher compared with control patients (median 33.96 vs. 24.41; *p* < 0.01, respectively); N gene Ct values was significantly higher compared with control patients (median 23.96 vs. 32.90; *p* < 0.01, respectively).

### Crude and independent factors associated with persistently PCR positive

The primary purpose of this study was to observe the risk factors related with occurrence of persistently viral RNA clearance. Among 41,390 enrolled patients, there were 1,023 patients who had viral RNA clearance ≥ 15 days after diagnosis onset. The matched patients had persistent positive viral detection results less than 8 days after diagnosis onset (*n* = 3061).

**Figure 2:**
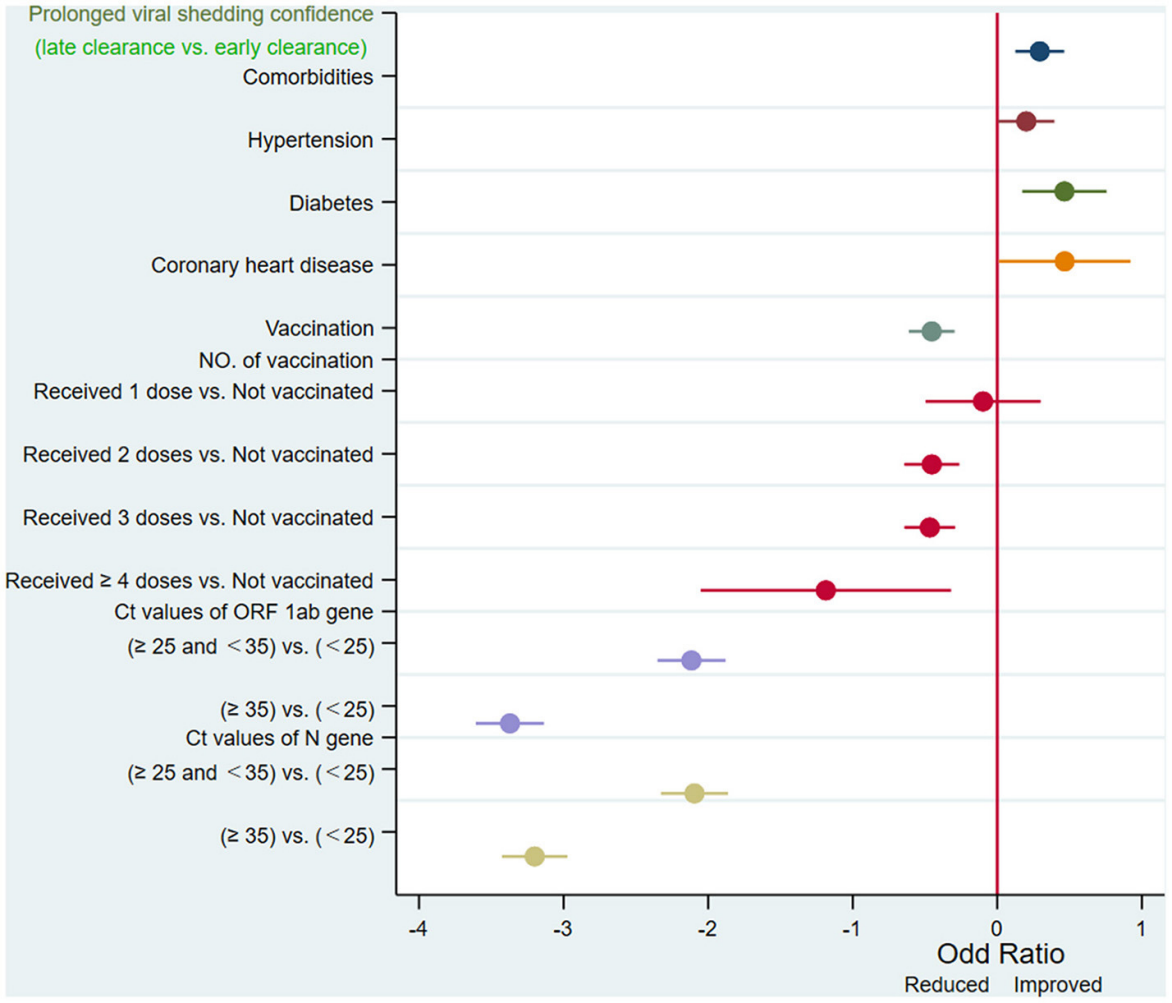
Factors associated with persistently PCR-positive patients at univariate regression analysis. Ct, cycle threshold; ORF 1ab gene, open reading frame 1ab; N gene, nucleocapsid protein gene.

Table 2 summarized the results of univariate analysis. We evaluated the effect of each factor on prolonged viral RNA clearance by logistic regression. Concomitant comorbidity (*p* = 0.001), hypertension (*p* = 0.043), diabetes (*p* = 0.002), coronary heart disease (*p* = 0.043), vaccine (*p* < 0.001), numbers of vaccine (*p* < 0.001), ORF 1ab gene (*p* < 0.001) and N gene Ct value (*p* < 0.001) on admission were significantly related to prolonged viral shedding (Table 2, Figure 2).

**Table 2:** Factors associated with persistently PCR-positive patients at univariate regression analysis.

**Determinants**	**Univariate models**	
	**OR (95% CI)**	***P-*value**
Comorbidities		
No	1 (ref)	
Yes	1.342 (1.133–1.589)	0.001
Hypertension		
No	1 (ref)	
Yes	1.222 (1.006–1.485)	0.043
Diabetes		
No	1 (ref)	
Yes	1.591 (1.188–2.131)	0.002
Coronary heart disease		
No	1 (ref)	
Yes	1.595 (1.011–2.514)	0.045
Vaccine		
No	1 (ref)	
Yes	0.635 (0.0.542–0.745)	<0.001
Vaccine		<0.001
No. vaccine		<0.001
Not vaccinated	1 (ref)	
Received 1 dose of vaccine	0.906 (0.609–1.350)	0.629
Completed 2 dose of vaccine	0.636 (0.525–0.769)	<0.001
Completed 3 dose of vaccine	0.627 (0.526–0.747)	<0.001
Completed ≥4 dose of vaccine	0.305 (0.128–0.726)	0.007
ORF 1ab ct value on admission		<0.001
<25	1 (ref)	
25–35	0.121 (0.095–0.153)	<0.001
≥35	0.034 (0.027–0.043)	<0.001
N ct value on admission		<0.001
<25	1 (ref)	
25-35	0.123 (0.098–0.155)	<0.001
>35	0.041 (0.032–0.051)	<0.001

Data are presented as OR (95% CI). OR, odds ratio; Ct, cycle threshold; ORF 1ab gene, open reading frame 1ab; N gene, nucleocapsid protein (N) gene; CI, confidence interval.

**Figure 3:**
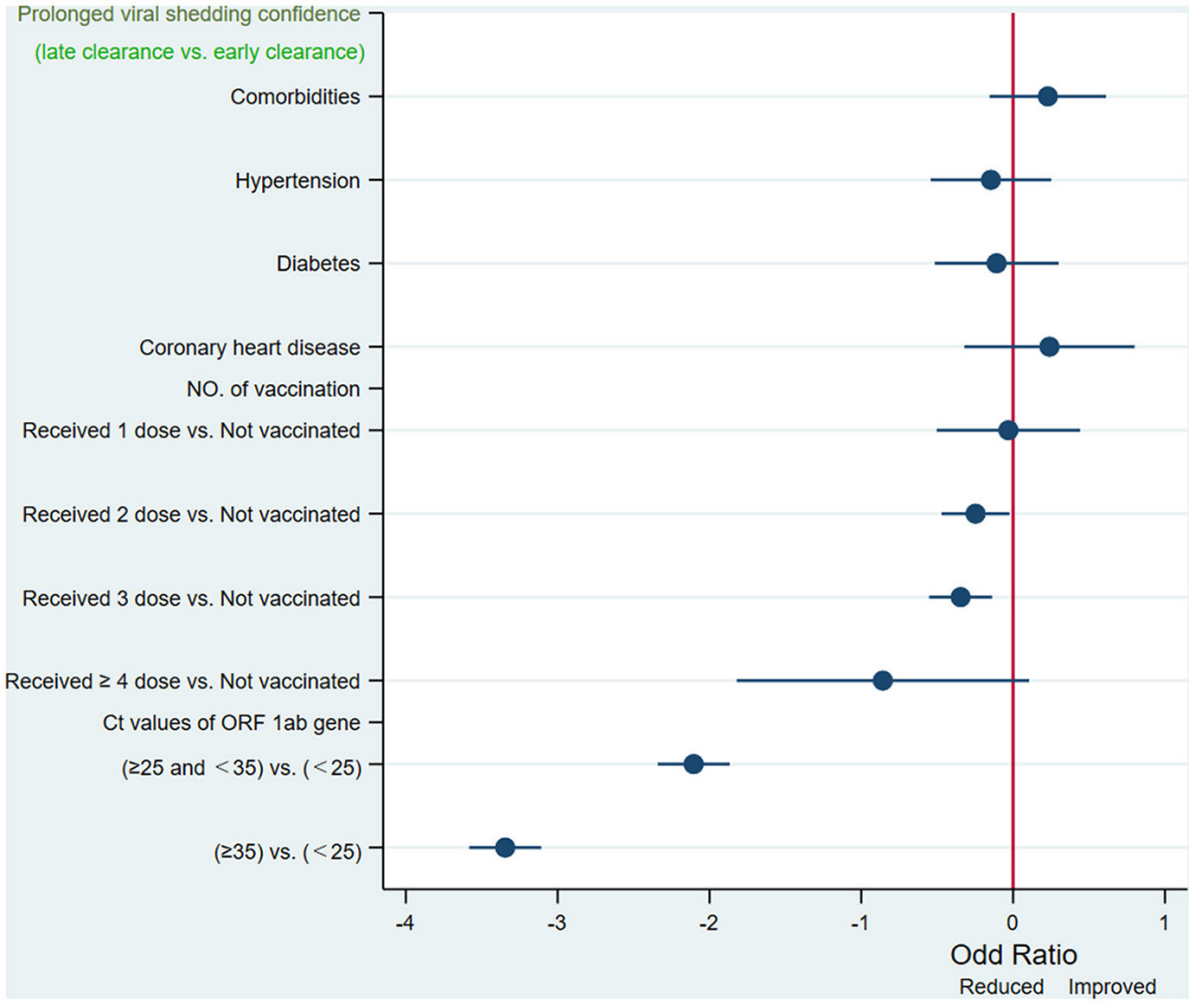
Independent predictors of persistently PCR-positive patients identified by unadjusted multivariate logistic regression models logistic regression. Ct, cycle threshold; ORF 1ab gene, open reading frame 1ab.

Multivariate logistic regression was then performed with the significant factors selected by unadjusted and adjusted univariate analysis. Multivariate binary logistic regression analysis model found that the doses of vaccination (2 doses, OR 0.781, *p* < 0.031; 3 doses, OR 0.708, *p* = 0.001), and ORF 1ab gene high (OR: 0.122, 95% CI: 0.096–0.155), and negative (OR: 0.035, 95% CI: 0.028–0.045). Ct values at admission, were independently associated with prolonged RT-PCR positive test results (Table 3, Figure 3).

**Table 3:** Independent predictors of persistently PCR-positive patients identified by unadjusted and adjusted multivariate logistic regression models.

**Determinants**	**Multivariate analysis**	***P*-value**
	**OR (95% CI)**	
Comorbidities	1.257 (0.856–1.846)	0.243
Hypertension	0.864 (0.581–1.285)	0.471
Diabetes	0.897 (0.597–1.349)	0.602
Coronary heart disease	1.271 (0.725–2.228)	0.403
NO. vaccine		0.010
Not vaccinated	1 (ref)	
Received 1 dose of vaccine	0.970 (0.604–1.556)	0.899
Received 2 dose of vaccine	0.781 (0.624–0.978)	0.031
Received 3 dose of vaccine	0.708 (0.575–0.871)	0.001
Received 4 dose of vaccine	0.424 (0.162–1.111)	0.081
ORF 1ab gene Ct value on admission		<0.001
<25	1 (ref)	<0.001
25≥ <35	0.122 (0.096–0.155)	<0.001
35 ≤	0.035 (0.028–0.045)	<0.001

OR, odds ratio; Ct, cycle threshold; ORF 1ab gene, open reading frame 1ab; N gene, nucleocapsid protein (N) gene; CI, confidence interval.

### GEE estimation of factors associated with time and RNA Ct value

**Figure 4:**
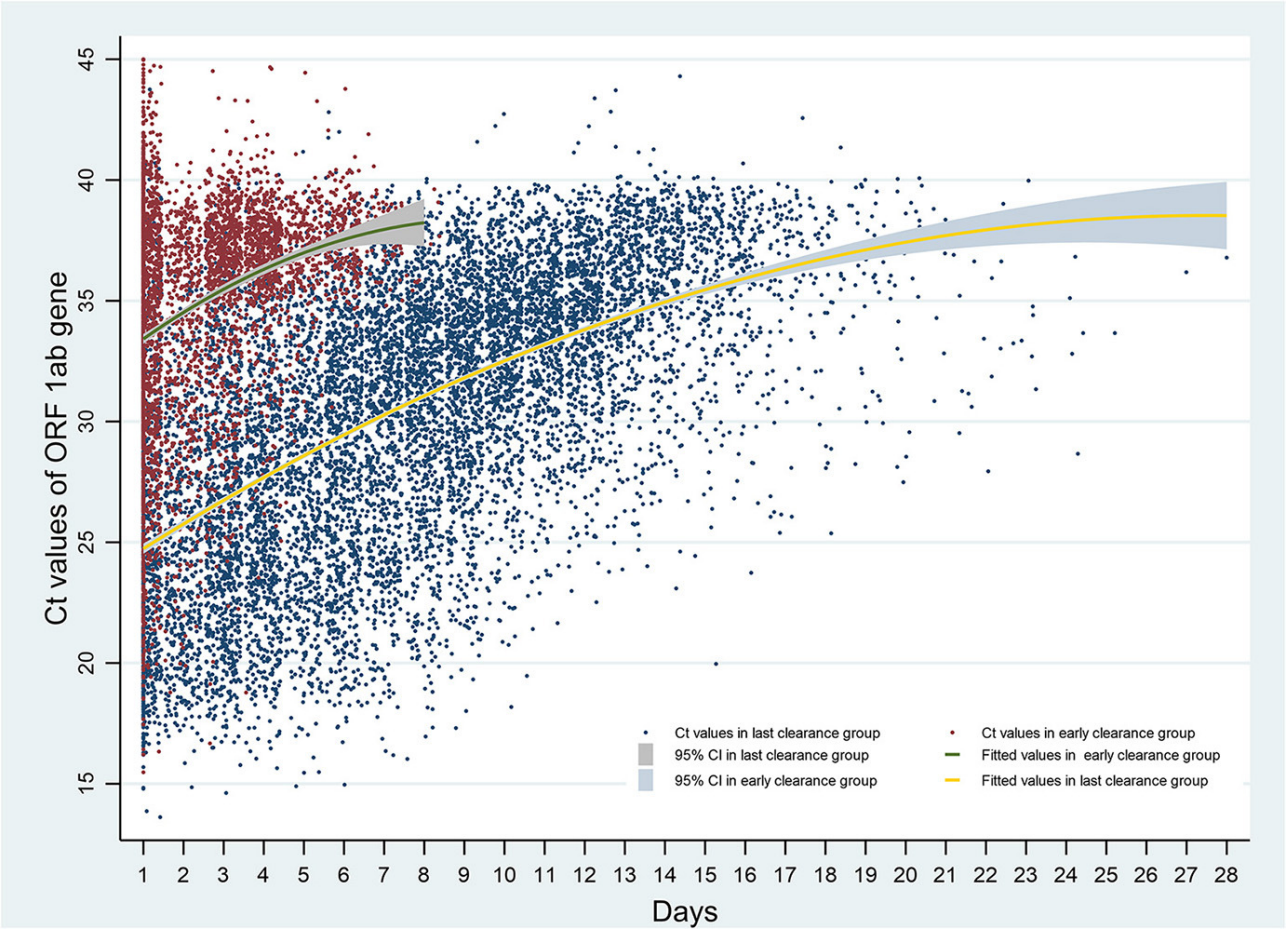
Ct values of ORF 1ab gene over time after admission. Ct, cycle threshold; ORF 1ab gene, open reading frame 1ab.

**Figure 5:**
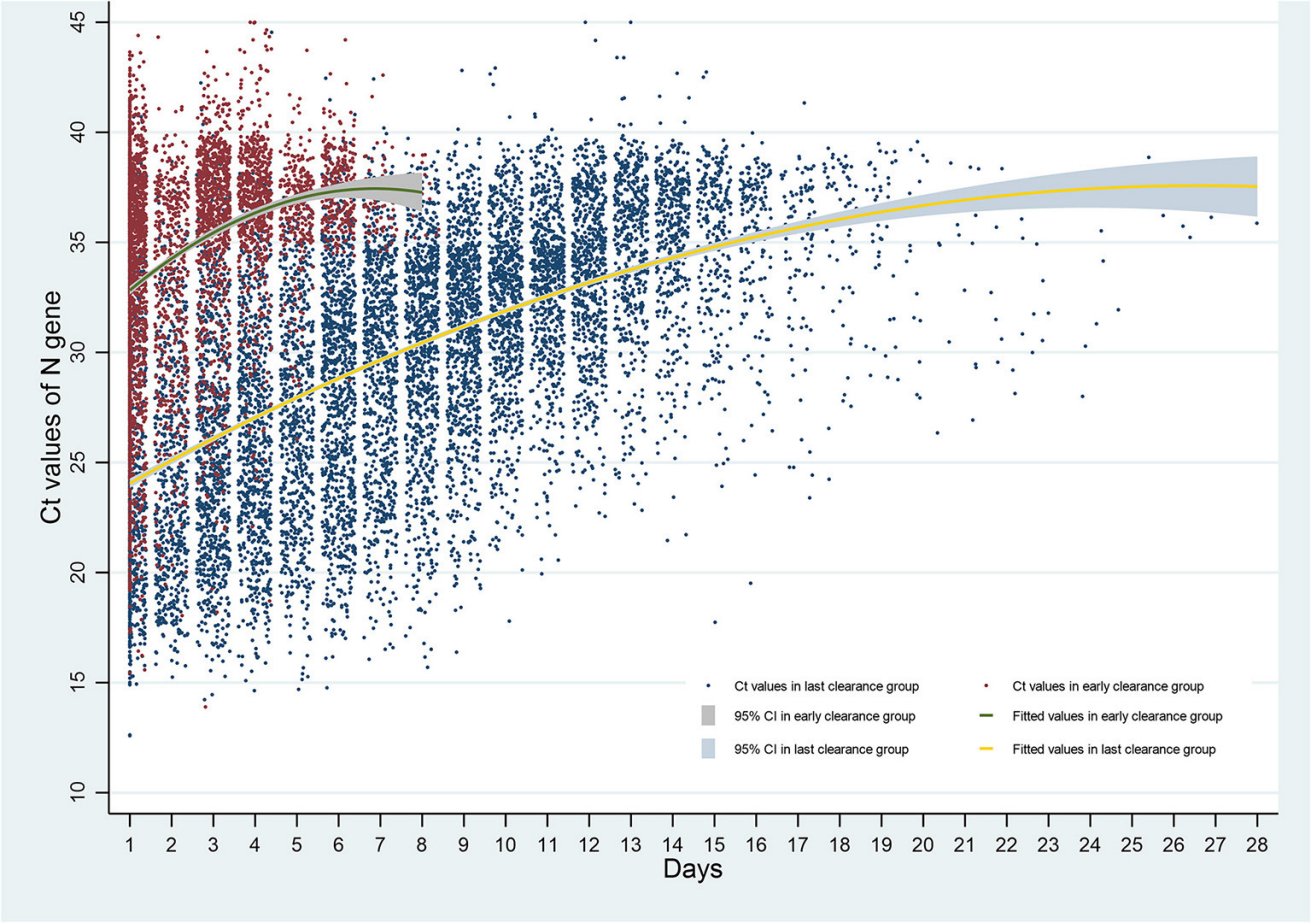
Ct values of N gene over time after admission. Ct, cycle threshold; N gene, nucleocapsid protein gene.

Figures 4, 5 show the increases of Ct values generally over time in both early and late-viral-RNA-clearance groups. Viral loads in nasopharyngeal swabs were estimated with Ct values by RT-PCR. For late-viral-RNA-clearance patients, the mean Ct values for RT-PCR on ORF 1ab gene was 33.59 (95%IC: 33.27–33.91), and on N gene was 32.85 (95%IC: 32.52–33.18; Tables 4, 5). Among prolonged-positive patients, terminal positive tests were a mean of 10.61 cycles and 10.62 cycles higher than first positive tests for ORF 1ab gene and N gene respectively. For early-viral-RNA-clearance patients' terminal and initial tests, terminal positive tests had a mean of 4.17 cycles and 8.39 cycles greater than initial positive tests for ORF 1ab gene and N gene respectively (Table 6). GEE estimation was undertaken in both groups respectively. The ORF gene and N gene CT values of patients with late-viral-RNA-clearance remained unchanged within 3 days, and showed progressively higher values with increasing days (β ranged over = 0.942–11.647 for ORF gene; 0.991–11.981 for N gene, *p* < 0.001; Tables 7, 8).

**Table 4:** Ct values of ORF 1ab gene over time after admission (Mean ± SE, 95% CI).

	**Late clearance**	**(95% CI)**	**Early clearance**	**(95% CI)**
	**Mean ±SE**		**Mean ±SE**	
Day 1	26.31 ± 0.21	25.90–26.72	33.64 ± 1.33	33.37–33.90
Day 2	26.23 ± 0.20	25.83–26.62	35.17 ± 0.18	34.82–35.52
Day 3	26.58 ± 0.17	26.24–26.91	36.38 ± 0.12	36.14–36.62
Day 4	27.25 ± 0.17	26.92–27.59	37.17 ± 0.10	36.96–37.37
Day 5	27.90 ± 0.18	27.55–28.25	37.66 ± 0.14	37.38–37.94
Day 6	29.09 ± 0.17	28.76–29.42	37.97 ± 0.11	37.76–38.18
Day 7	30.18 ± 0.17	29.85–30.51	38.21 ± 0.18	37.85–38.57
Day 8	31.01 ± 0.15	30.70–31.31	37.81 ± 0.41	37.01–38.60
Day 9	32.01 ± 0.14	31.73–32.29		
Day 10	33.08 ± 0.13	32.82–33.34		
Day 11	34.00 ± 0.13	33.74–34.26		
Day 12	34.38 ± 0.13	34.12–34.64		
Day 13	35.44 ± 0.16	35.12-−5.75		
Day 14	36.03 ± 0.17	35.70–36.37		
Day 15	36.62 ± 0.21	36.20–37.04		
Day 16	36.64 ± 0.24	36.18–37.11		
Day 17	36.63 ± 0.37	35.91–37.35		
Day 18	36.72 ± 0.48	35.78–37.67		
Day 19	36.92 ± 0.53	35.88–37.95		
Day 20	36.40 ± 0.76	34.90–37.90		
Day 21	36.49 ± 1.04	34.44–38.54		
Day 22	35.66 ± 0.96	33.78–37.53		
Day 23	36.32 ± 0.95	34.45–38.18		
Day 24	35.07 ± 1.04	33.03–37.11		
Day 25	37.59 ± 1.13	35.37–39.81		
Day 26	38.11 ± 0.42	37.28–38.94		
Day 27	37.66 ± 0.34	37.00–38.33		
Day 28	37.08 ± 0.18	36.72–37.44		

CI, confidence interval; SE, standard error; Ct, cycle threshold. ORF 1ab gene, open reading frame 1ab.

**Table 5:** Ct values of N gene over time after admission (Mean ± SE, 95% CI).

	**Late clearance**	**(95% CI)**	**Early clearance**	**(95% CI)**
	**Mean ±SE**		**Mean ±SE**	
Day 1	25.67 ± 0.21	25.26–26.09	33.03 ± 0.12	32.79–33.27
Day 2	25.63 ± 0.20	25.23–26.03	35.39 ± 0.20	34.99–35.79
Day 3	25.99 ± 0.18	25.64–26.33	37.06 ± 0.15	36.77–37.34
Day 4	26.66 ± 0.18	26.32–27.01	38.30 ± 0.15	38.00–38.59
Day 5	27.25 ± 0.18	26.91–27.60	39.77 ± 0.20	39.38–40.16
Day 6	28.46 ± 0.17	28.13–28.78	40.29 ± 0.21	39.88–40.69
Day 7	29.57 ± 0.17	29.24–29.91	41.31 ± 0.28	40.76–41.86
Day 8	30.30 ± 0.16	29.99–30.60	41.42 ± 0.61	40.24–42.61
Day 9	31.33 ± 0.15	31.04–31.61		
Day 10	32.33 ± 0.14	32.06–32.60		
Day 11	33.26 ± 0.14	32.99–33.53		
Day 12	33.83 ± 0.14	33.56–34.10		
Day 13	34.96 ± 0.17	34.63–35.28		
Day 14	35.39 ± 0.17	35.06–35.73		
Day 15	35.67 ± 0.21	35.25–36.09		
Day 16	35.84 ± 0.25	35.35–36.33		
Day 17	35.79 ± 0.36	35.08–36.51		
Day 18	36.24 ± 0.43	35.40–37.08		
Day 19	36.32 ± 0.48	35.37–37.27		
Day 20	35.61 ± 0.81	34.02–37.20		
Day 21	35.50 ± 0.98	33.59–37.41		
Day 22	33.90 ± 0.93	32.08–35.73		
Day 23	34.14 ± 0.89	32.40–35.87		
Day 24	33.10 ± 1.12	30.90–35.29		
Day 25	36.67 ± 1.16	34.40–38.95		
Day 26	36.48 ± 0.45	35.60–37.37		
Day 27	37.62 ± 0.27	37.13–38.18		
Day 28	36.29 ± 0.13	36.04–36.55		

CI, confidence interval; SE, standard error; Ct, cycle threshold; N gene, nucleocapsid protein gene.

**Table 6:** Mean Ct values for RT-PCR on ORF 1ab gene and N gene during viral RNA clearance.

	**Late clearance**	**Early clearance**
ORF 1ab, Mean ± SE (95% CI)	33.59 ± 0.16 (33.27–33.91)	38.07 ± 0.11 (37.85–38.29)
N, Mean ± SE (95% CI)	32.85 ± 0.17 (32.52–33.18)	38.32 ± 0.12 (38.52–33.18)

CI, confidence interval; SE, standard error; Ct, cycle threshold; N gene, nucleocapsid protein gene; ORF 1ab gene, open reading frame 1ab.

**Table 7:** Estimated factors associated with Ct values of ORF 1ab gene with times by GEE.

	**Late clearance**		**Early clearance**	
	**β (95% CI)**	***P***	**β (95% CI)**	***P***
Intercept	26.301		33.556	
Time				
Day 1	1 (ref)		1 (ref)	
Day 2	−0.126 (−0.539 to 0.286)	0.548	2.215 (1.807–2.624)	<0.001
Day 3	0.263 (−0.207 to 0.734)	0.273	3.481 (3.134–3.828)	<0.001
Day 4	0.942 (0.451–1.432)	<0.001	4.561 (4.168–4.594)	<0.001
Day 5	1.591 (1.055–2.127)	<0.001	5.815 (5.334–6.296)	<0.001
Day 6	2.779 (2.242–3.317)	<0.001	6.189 (5.675–6.704)	<0.001
Day 7	3.872 (3.317–4.427)	<0.001	6.875 (6.198–7.553)	<0.001
Day 8	4.691 (4.151–5.230)	<0.001	6.993 (5.965–8.020)	<0.001
Day 9	5.685 (5.162–6.208)	<0.001		
Day 10	6.760 (6.273–7.247)	<0.001		
Day 11	7.670 (7.183–8.157)	<0.001		
Day 12	8.048 (7.579–8.518)	<0.001		
Day 13	9.100 (8.582–9.619)	<0.001		
Day 14	9.692 (9.164–10.219)	<0.001		
Day 15	10.230 (9.648–10.812)	<0.001		
Day 16	10.240 (9.636–10.843)	<0.001		
Day 17	10.194 (9.377–11.012)	<0.001		
Day 18	10.249 (9.217–11.28)	<0.001		
Day 19	10.429 (9.323–11.535)	<0.001		
Day 20	9.922 (8.380–11.464)	<0.001		
Day 21	9.936 (7.861–12.012)	<0.001		
Day 22	9.128 (7.224–11.031)	<0.001		
Day 23	9.820 (7.909–11.732)	<0.001		
Day 24	8.629 (6.516–10.741)	<0.001		
Day 25	11.049 (8.699–13.40)	<0.001		
Day 26	11.647 (10.772–12.521)	<0.001		
Day 27	11.097 (10.373–11.821)	<0.001		
Day 28	10.587 (10.073–11.102)	<0.001		

CI, confidence interval; SE, standard error; GEE, generalized estimating equations.

**Table 8:** Estimated factors associated with Ct values of N gene with times by GEE.

	**Late clearance**		**Early clearance**	
	**β (95% CI)**	***P*-value**	**β (95% CI)**	***P*-value**
Intercept	26.301		33.029	
time				
1	1 (ref)		1 (ref)	
2	−0.039 (−0.456 to 0.378)	0.855	2.360 (1.939–2.782)	<0.001
3	0.315 (−0.159 to 0.790)	0.193	4.030 (3.674–4.386)	<0.001
4	0.991 (0.488–1.494)	<0.001	5.273 (4.881–4.594)	<0.001
5	1.581 (1.04–2.122)	<0.001	5.815 (5.334–5.664)	<0.001
6	2.784 (2.243–3.325)	<0.001	6.738 (6.273–7.203)	<0.001
7	3.902 (3.344–4.460)	<0.001	8.279 (7.659–8.898)	<0.001
8	4.625 (4.08–5.168)	<0.001	8.396 (7.184–9.607)	<0.001
9	5.654 (5.132–6.176)	<0.001		
10	6.662 (6.170–7.153)	<0.001		
11	7.584 (7.093–8.076)	<0.001		
12	8.155 (7.673–8.636)	<0.001		
13	9.284 (8.754–9.813)	<0.001		
14	9.722 (9.190–10.253)	<0.001		
15	9.994 (9.412–10.577)	<0.001		
16	10.168 (9.533–10.804)	<0.001		
17	10.120 (9.307–10.934)	<0.001		
18	10.565 (9.615–11.515)	<0.001		
19	10.648 (9.605–11.691)	<0.001		
20	9.938 (8.301–11.575)	<0.001		
21	9.830 (7.87–11.790)	<0.001		
22	8.231 (6.375–10.088)	<0.001		
23	8.466 (6.691–10.240)	<0.001		
24	7.422 (5.193–9.651)	<0.001		
25	11.002 (8.676–13.328)	<0.001		
26	10.810 (9.841–11.779)	<0.001		
27	11.981 (11.297–12.664)	<0.001		
28	10.620 (10.125–11.115)	<0.001		

CI, confidence interval; SE, standard error.

## Discussion

Despite the amount of research published during the continuous COVID-19 pandemic, there is still a dearth of literature about factors possibly influencing prolonged SARS-CoV-2 Omicron variant shedding. The present study was conducted to identify clinical characteristics and factors associated with prolonged RT-PCR positivity for Omicron variant patients. We classified patients based on their length of RNA shedding, and observed that 25.05% of the 1,023 study patients required more than 15 days to achieve a negative viral RNA test. Moreover, 3,061 individuals were matched by the timing of diagnosis, sex, ages, wards, with viral RNA clearance occurred less than or equal to 8 days. Our findings showed the median interval from diagnosis to SARS-CoV-2 nucleic acid conversion in prolonged positivity patients was 16 days, shorter than the 21 days in many previous reports (8, 19). Bennasrallah et al. (20) found that negative conversion of SARS-CoV-2 RNA occurred 20 days (IQR: 17–32 days) from the first positive RT-PCR test. Another study in Italy reported a longer median duration of the nucleic acid conversion negative of 31 days (IQR 24–41 days) (21). Ling et al. (6) found the median time from the onset of symptoms to first negative conversion results of oropharyngeal swabs in convalescent patients was 9.5 (6.0–11.0) days. The discrepancies between studies might attribute to differences in disease severity, sampling methods, as well as the varieties of virus. These results might suggest that Omicron variant was relatively less severe than previous strains (22).

Our study population have a mean age of 50 years and included a higher proportion (61.4%) of male. These demographic characteristics were comparable to the patients in studies reported in 2020 (23). As control patients were matched by sex and age, there were no gender or sex difference between the two groups. However, a study conducted by cox regression multivariate analysis revealed that growth of age was correlated to later RT-PCR conversion (11).

Our finding showed that 82.3% of the reported COVID-19 cases were asymptomatic, while in studies reported in 2020, all patients had at least one symptom and 34.2% of those developed severe COVID-19 (8). COVID-19-positive cases with at least one chronic comorbidity had higher percentage in the prolonged-viral-RNA-shedding group. Hypertension, diabetes, and coronary heart disease were the most frequent chronic comorbidities reported in our COVID-19 Omicron variant population. However, these comorbidities were not significant risk factors in the logistic regression model.

On admission, almost 83% were asymptomatic COVID-19 cases, while 19.7% were at least one symptom. Interesting, our results showed there were no different in a duration of viral shedding between asymptomatic and symptomatic patients. A previous study on 70 patients hospitalized with COVID-19 demonstrated clinical classification (symptomatic and asymptomatic) was not a significant predictor for the time to RT-PCR conversion (24). Carmo et al. (25) indicated that prolonged viral RNA is not necessarily related to severe disease, as mild illness patients discharged home took longer to convert negative than inpatients.

There have been no specific antiviral drugs for SARS-CoV-2. In our study, the percentage of patients with vaccination at least one dose was lower in late-clearance group than early-clearance group. Multivariate logistic regression showed vaccination, especially 2 or 3 doses of vaccination, was significant related to prolonged nucleic acid conversion time. Wu et al. (26) conducted a retrospective cohort, enrolling 142 patients with no or mild symptoms, to evaluate whether vaccination could improve the disease course of SARS-CoV-2 Omicron variant. The result found that but patients with vaccination had shorter time to target cycle threshold value.

Additionally, Ct values may be an important proxy for infectivity. Our results suggest that viral-RNA Ct values on admission were supported as an independent factor related to prolonged RT-PCR positivity. Patients were at a higher risk of having a higher viral load than control patients, during a prolonged period of time. And Ct values of RT-PCR test in throat swab peaked at around 2–3 days after admission. Pan et al. (27) supported viral loads in throat swab and sputum samples peaked at around 5–6 days after symptom onset.

This study had some limitations. First, although viral RNA was detected in most of the studies, viral RNA shedding cannot exactly represent viral shedding. So far, viable and nonviable viruses on nasopharyngeal swab cannot be distinguished by RT-PCR for SARS-CoV-2 RNA. Second, the study ignored patients who recovered PCR negative 9-14 days after initial PCR positive.

## Conclusion

In conclusion, prolonged SARS-CoV-2 RNA shedding was independently associated with vaccination and ORF 1ab gene viral-RNA Ct values. These results emphasized that hospital admission and vaccination should be started as soon as possible in patients with COVID-19. Understanding the virological dynamics during the process of illness should be helpful in the clinical management of patients with COVID-19.

## Data availability statement

The raw data supporting the conclusions of this article will be made available by the authors, without undue reservation.

## Ethics statement

The studies involving human participants were reviewed and approved by the Longhua Hospital, Shanghai University of Traditional Chinese Medicine Ethical Review Authority (DNR 2020-02150) and registered in the Chinese Clinical Trial Registry (ChiCTR2200060472). Written informed consent to participate in this study was provided by the participants' legal guardian/next of kin.

## Author contributions

BF, HH, and SZ study conception and design. DS and MC acquisition of clinical and drug concentration data. WL, LS, XX, YP, CC, YS, and HY analysis and interpretation of data. WZ, GW, and YG wrote manuscript. All authors contributed to the article and approved the submitted version.

## Funding

Funding for this study was provided by National Key R&D Program of China (2018YFC1705900); State Administration of Traditional Chinese Medicine, traditional Chinese medicineon prevention and treatment of novel Coronavirus pneumoniaemergency and special project (2022ZYLCYJ05-3 and 2022ZYLCYJ05-4); and Shanghai University of Traditional Chinese Medicine, traditional Chinese medicine on prevention and treatment of novel Coronavirus pneumonia emergency and special project (2022YJ-03 and 2022YJ-06).

## Conflict of interest

The authors declare that the research was conducted in the absence of any commercial or financial relationships that could be construed as a potential conflict of interest.

## Publisher's note

All claims expressed in this article are solely those of the authors and do not necessarily represent those of their affiliated organizations, or those of the publisher, the editors and the reviewers. Any product that may be evaluated in this article, or claim that may be made by its manufacturer, is not guaranteed or endorsed by the publisher.
